# Perinatal characteristics and neonatal outcomes of singletons and twins in Chinese very preterm infants: a cohort study

**DOI:** 10.1186/s12884-023-05409-8

**Published:** 2023-02-01

**Authors:** Min Yang, Lingyu Fang, Yanchen Wang, Xiaoying Li, Yun Cao, Jianhua Sun, Joseph Ting, Xiafang Chen, Xiaobo Fan, Jiale Dai, Xiaomei Tong, Dongmei Chen, Jimei Wang, the Chinese Neonatal Network, Shoo K. Lee, Chao Chen, Lizhong Du, Wenhao Zhou, Falin Xu, Xiuying Tian, Huayan Zhang, Yong Ji, Zhankui Li, Jingyun Shi, Xindong Xue, Chuanzhong Yang, Sannan Wang, Ling Liu, Xirong Gao, Hui Wu, Changyi Yang, Shuping Han, Ruobing Shan, Hong Jiang, Gang Qiu, Qiufen Wei, Rui Cheng, Wenqing Kang, Mingxia Li, Yiheng Dai, Lili Wang, Jiangqin Liu, Zhenlang Lin, Yuan Shi, Xiuyong Cheng, Jiahua Pan, Qin Zhang, Xing Feng, Qin Zhou, Long Li, Pingyang Chen, Ling Yang, Deyi Zhuang, Yongjun Zhang, Jinxing Feng, Li Li, Xinzhu Lin, Yinping Qiu, Kun Liang, Li Ma, Liping Chen, Liyan Zhang, Hongxia Song, Zhaoqing Yin, Mingyan Hei, Huiwen Huang, Jie Yang, Dong Li, Guofang Ding, Qianshen Zhang, Xiaolu Ma

**Affiliations:** 1 Division of Neonatology, Obstetrics and Gynecology Hospital of Fudan University, No. 419, Fangxie Road, Shanghai, 200011 China; 2 Division of Neonatology, QuanZhou Women’s and Children’s Hospital, No. 700, Fengze Road, Fujian, 362000 China; 3 NHC Key Laboratory of Neonatal Diseases, Fudan University, Children’s Hospital of Fudan University, Shanghai, 201102 China; 4 Division of Neonatology, Children’s Hospital Affiliated to Shandong University, Jinan, 250022 China; 5 Division of Neonatology, Children’s Hospital of Fudan University, Shanghai, 201102 China; 6 Division of Neonatology, Shanghai Children’s Medical Center, Shanghai Jiao Tong University School of Medicine, Shanghai, 200127 China; 7 Department of Pediatrics, University of Alberta, EdmontonAlberta, T6G 1K8 Canada

**Keywords:** Neonatal outcome, Twins, Singletons, Very preterm birth

## Abstract

**Background:**

The prevalence of preterm birth has been rising, and there is a paucity of nationwide data on the perinatal characteristics and neonatal outcomes of twin deliveries of very preterm infants (VPIs) in China. This study compared the perinatal characteristics and outcomes of singletons and twins admitted to neonatal intensive care units (NICUs) in China.

**Methods:**

The study population comprised all infants born before 32 weeks in the Chinese Neonatal Network (CHNN) between January 2019 and December 2019. Three-level and population-average generalized estimating equation (GEE)/alternating logistic regression (ALR) models were used to determine the association of twins with neonatal morbidities and the use of NICU resources.

**Results:**

During the study period, there were 6634 (71.2%) singletons and 2680 (28.8%) twins, with mean birth weights of 1333.70 g and 1294.63 g, respectively. Twins were significantly more likely to be delivered by caesarean section (*p* < 0.01), have antenatal steroid usage (*p* = 0.048), have been conceived by assisted reproductive technology (ART) (*p* < 0.01), have a higher prevalence of maternal diabetes (*p* < 0.01) and be inborn (*p* < 0.01) than singletons. In addition, twins had a lower prevalence of small for gestational age, maternal hypertension, and primigravida mothers than singletons (all *p* < 0.01). After adjusting for potential confounders, twins had higher mortality rates (adjusted odds ratio [AOR] 1.28, 95% confidence interval [CI] 1.10–1.49), higher incidences of short-term composite outcomes (AOR 1.28, 95% CI 1.09–1.50), respiratory distress syndrome (RDS) (AOR 1.30, 95% CI 1.12–1.50), and bronchopulmonary dysplasia (BPD) (AOR 1.10, 95% CI 1.01–1.21), more surfactant usage (AOR 1.22, 95% CI 1.05–1.41) and prolonged hospital stays (adjusted mean ratio 1.03, 95% CI 1.00–1.06), compared to singletons.

**Conclusion:**

Our work suggests that twins have a greater risk of mortality, a higher incidence of RDS and BPD, more surfactant usage, and longer NICU stays than singletons among VPIs in China.

## Background

Over the past decade, the rate of very preterm twins has been increasing worldwide mainly due to the development of assisted reproductive technology (ART) and increased maternal childbearing age [1–7]. Data from the Australian and New Zealand Neonatal Network showed that the incidence of twins in extremely preterm infants increased from 21.6% in 1995–1999 to 25.6% in 2005–2009 [5]. Studies have revealed controversial findings regarding the neonatal outcomes of multiple pregnancies. Some studies have reported an increased risk of infant mortality and morbidity associated with twin pregnancies [4, 5, 7–9]. In contrast, other studies did not reveal such differences in multiples compared with singletons [10, 11]. There is a lack of data on the morbidity and mortality of preterm multiples compared with singletons in China, which is important for counselling and decision-making in obstetric practice.

The Chinese Neonatal Network (CHNN) was founded in 2018 and comprises 58 major tertiary hospitals from 25 provinces across China [12]. The purpose of this study was to explore the perinatal characteristics and neonatal outcomes for twins compared with singletons among VPIs in China.

## Methods

### Data source

This was a retrospective analysis of a prospectively collected cohort study of neonates delivered at < 32 weeks gestation from the CHNN. The CHNN is a collaborative network of 58 major tertiary hospitals providing care management for high-risk neonates, including four national children’s medical centres, four regional children’s medical centres, thirty provincial perinatal or children’s medical centres and nineteen major referral centers in large cities in China. Among them, one hospital was excluded due to incomplete data, and ultimately 57 hospitals collecting full-year data on VPIs admitted to the neonatal unit in 2019 were included in this study. These hospitals accounted for approximately 5% of all VPIs in China. These hospitals included all government-designated neonatal centres of excellence in China. The median number of neonatal intensive care unit (NICU) beds was 40 (interquartile range [IQR], 30–62), and the median number of intermediate-level and continuing care neonatal beds was 66 (IQR, 40–91). For perinatal centres, the median number of annual deliveries was 10 280 (IQR, 6273–15 423). The median number of full-time equivalent neonatologists was 19 (IQR, 12–27), and the median number of NICU nurses was 42 (IQR, 30–65). The CHNN maintains a standardized clinical database to monitor the outcomes and care practices of NICUs in Chinese tertiary hospitals [12].

Trained abstractors prospectively collected data from NICU electronic medical records using a standard manual of variable definitions. A customized database with built-in error checking was applied during data entry. Data were later electronically submitted to the CHNN coordinating centre in the Children’s Hospital of Fudan University with patient anonymity. The accuracy of the data collection was monitored by investigators, and rigorous quality control procedures were performed at each site [13].

### Study population

All infants in our study were delivered at less than 32 weeks gestation and admitted to CHNN hospitals from January 2019 to December 2019. Infants with major congenital anomalies, chromosomal defects, and those from multiple pregnancies other than twin pregnancies were excluded. The study population was grouped by plurality (singletons, twins). Readmissions and transfers between participating hospitals were recorded as data from the same infants. Data from infants who were stillborn, died in the delivery room, or were transferred to nonparticipating hospitals within 24 h after birth were not recorded in the database.

### Outcomes and definitions

The primary outcome was a composite of mortality or any severe neonatal morbidity and short-term composite outcomes. Severe neonatal morbidity was defined as necrotizing enterocolitis (NEC) ≥ stage 2, bronchopulmonary dysplasia (BPD), severe neurological injury (severe intraventricular haemorrhage [IVH] and cystic periventricular leukomalacia [cPVL]), severe retinopathy of prematurity (ROP), or sepsis. Necrotizing enterocolitis (NEC) was defined according to Bell’s criteria [14, 15]. BPD was defined as a requirement for respiratory support at 36 weeks postmenstrual age or at discharge/transfer/death if before 36 weeks postmenstrual age [16]. Severe IVH was defined as grade 3 or 4 IVH according to Papile’s criteria [17]. cPVL was defined as the presence of periventricular cysts on cranial ultrasound or MRI. Severe ROP was defined as grade 3 or 4 ROP according to the international classification of ROP [18]. Sepsis was defined as a positive blood or cerebrospinal fluid culture and antibiotic therapy or the intent of providing antibiotic therapy for ≥ 3 days [19]. Short-term composite outcomes included early death and neonatal respiratory distress syndrome (RDS). Early death was defined as death occurring less than 7 days after birth. RDS was defined according to a combination of clinical signs and symptoms, laboratory analysis and chest X-ray (CXR) [20]. In addition, the secondary outcome was the use of NICU resources, including the length of NICU stay, surfactant use, breastfeeding, exclusive breastfeeding, and the duration of invasive ventilation. Breastfeeding was defined as an infant receiving their mother’s own milk, independent of the addition of formula or other foods and/or drinks. Exclusive breastfeeding was defined as an infant receiving no foods or drinks other than their mother’s own milk.

### Definitions of other covariates

Gestational age (GA) was recorded using the hierarchy of the best obstetric estimate based on prenatal ultrasound, menstrual history, obstetric examination, or all three. If the obstetric estimate was not available or was different from the postnatal estimate of gestation by more than two weeks, gestational age was estimated using the Ballard Score [21]. Small for gestational age (SGA) was defined as a birth weight < 10th percentile for the gestational age according to the Chinese neonatal birth weight values [22]. Prenatal care was defined as ≥ 1 pregnancy-related hospital visit during pregnancy. ART was defined as the use of hormonal therapy, artificial insemination, or any method of in vitro fertilization.

### Ethics approval

The conduction of this study was approved by the ethics committee of the Children’s Hospital of Fudan University (Identifier: 2018–296) and recognized by all participating NICUs for the development, compilation, data transfer, hosting, and analysis of the CHNN dataset. Individual consent for this retrospective analysis was waived in all 57 tertiary-level NICUs because this study did not directly intervene in the diagnosis and treatment of individual patients. This study was conducted in accordance with the Declaration of Helsinki (as revised in 2013).

### Statistical analysis

Perinatal characteristics and neonatal outcomes of singletons and twins were described by descriptive statistics. We also reported the means [standard deviations (SDs)] or medians [interquartile ranges (IQRs)] for continuous variables depending on normality. Chi-square tests, Student’s t tests or Mann‒Whitney U tests were applied to compare infants and maternal characteristics between singletons and twins as appropriate.

Three-level and population-average generalized estimating equation (GEE)/alternating logistic regression (ALR) models were used to determine the association of twins with neonatal morbidities and the use of NICU resources [23–26]. Adjusted odds ratios were reported after controlling for level-1 infant characteristics (gestational age, sex, SGA, birthplace) and level-2 maternal information (maternal age, antenatal steroid usage, hypertension, diabetes, c-section, ART, primigravida status). To account for the level-3 NICU cluster effect, an exchangeable correlation matrix was used in the multiple GEE/ALR regressions. Stratified analysis was carried out between infants with a GA < 28 weeks and those with a GA of 28–31 weeks to assess whether GA modified the impact of twins on neonatal morbidity and the use of NICU resources. All statistical analyses with data management were conducted by SAS version 9.4. The significance level of all hypothesis tests was 0.05 with a two-sided test.

## Results

### Study population

**Fig. 1:**
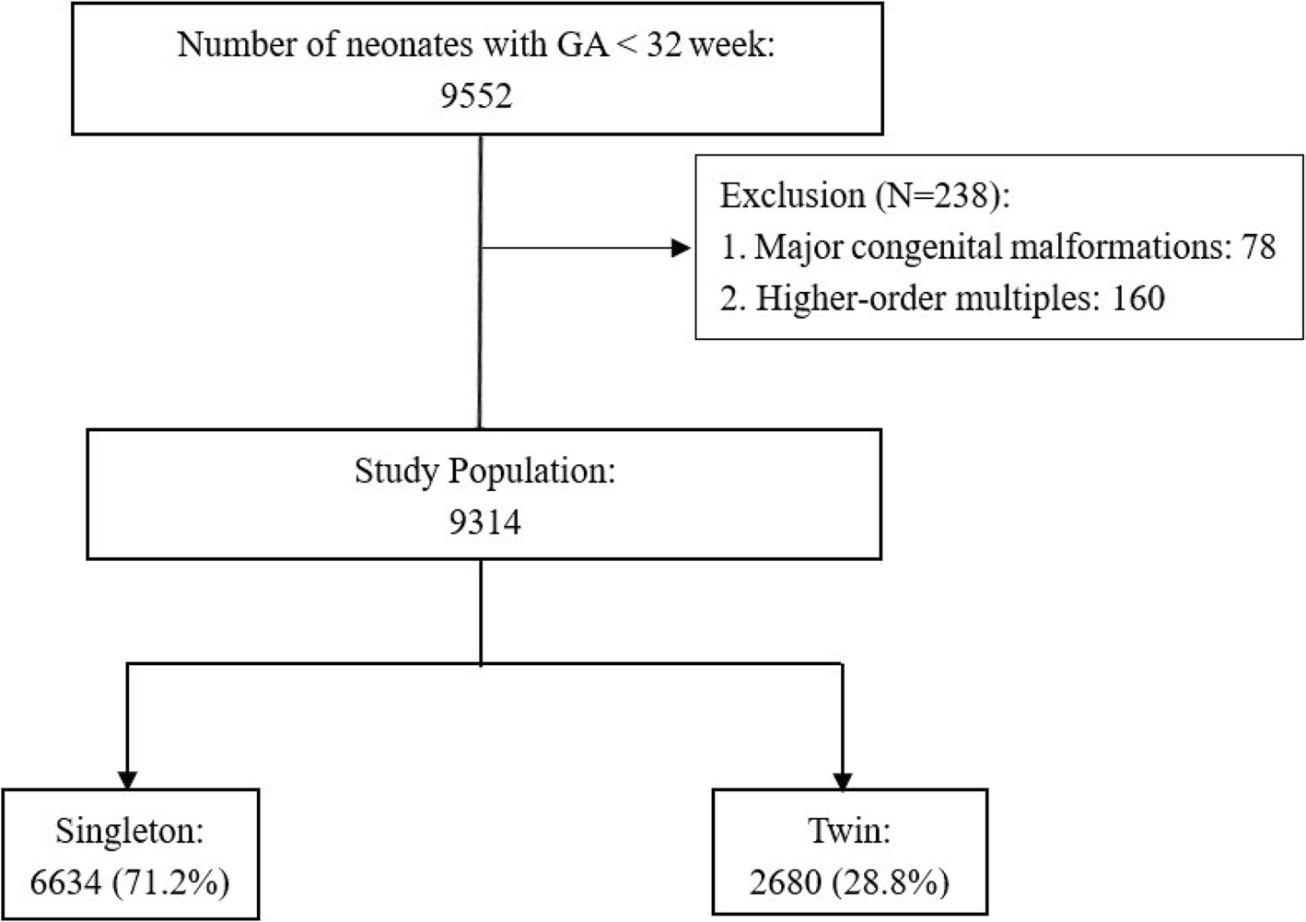
Flow diagram of the study participants

During the study period, 9552 infants were admitted to 57 neonatal intensive care units (NICUs). Among them, 78 infants had congenital anomalies or chromosomal defects, 160 infants were from higher-order multiple pregnancies (triplets and quadruplets), and 9314 infants met the inclusion criteria (6634 singletons (71.2%) and 2680 twins (28.8%)) (Fig. 1). Among them, 302 infants died after active medical treatments in the NICU, 61 infants died after palliative care, and 780 infants were discharged against medical advice. The median (IQR) gestational age for twins was lower than that of singletons, with a median of 29.7 (28.1, 31.0) weeks of GA for twins and 30.0 (28.4, 31.0) weeks of GA for singletons (*P* < 0.01). Moreover, the mean (SD) birth weight for twins was lower than that of singletons, with a mean of 1294.63 (313.99) g for twins and 1333.70 (323.21) g for singletons. There were 3832 male infants (57.9%) from singleton pregnancies and 1439 male infants (53.7%) from twin pregnancies.

### Basic perinatal characteristics

Twins were significantly more likely to have received ART (39.5% vs. 7.3%, *p* < 0.01), were born to mothers with diabetes (19.7% vs. 16.1%, *p* < 0.01), had antenatal steroid usage (77.0% vs. 74.9%, *p* = 0.048), were delivered by caesarean delivery (C-section) (59.6% vs. 52.5%, *p* < 0.01), and were inborn (66.4% vs. 62.6%, *p* < 0.01) than singletons. In addition, twins had a lower prevalence of small for gestational age (6.2% vs. 8.3%, *p* < 0.01), maternal hypertension (10.3% vs. 22.3%, *p* < 0.01) and primigravida mothers (46.2% vs. 53.0%, *p* < 0.01) than singletons. There were no significant differences in prenatal visits > 1 and Apgar scores < 7 at 5 min between these two groups (Table 1).

**Table 1:** Comparison of baseline perinatal characteristics in singleton and twin pregnancies

Outcome	Singleton	Twin	Overall	*P* value
**N**	**6634**	**2680**	**9314**	
**Maternal Information**				
Maternal Age, Mean (SD)	31.13 (5.08)	30.60 (4.72)	30.98 (4.98)	< 0.01
Antenatal Steriod Usage, N (%)	4470/5967 (74.9%)	1873/2434 (77.0%)	6343/8401 (75.5%)	0.048
Hypertension, N (%)	1456/6523 (22.3%)	269/2617 (10.3%)	1725/9140 (18.9%)	< 0.01
Diabetes, N (%)	1047/6507 (16.1%)	516/2615 (19.7%)	1563/9122 (17.1%)	< 0.01
Prenatal Visit > 1, N (%)	6307/6375 (98.9%)	2579/2606 (99.0%)	8886/8981 (98.9%)	0.9
Cesarean section, N (%)	3466/6596 (52.5%)	1592/2670 (59.6%)	5058/9266 (54.6%)	< 0.01
ART, N (%)	486/6634 (7.3%)	1059/2680 (39.5%)	1545/9314 (16.6%)	< 0.01
Primi-Gravida, N (%)	3490/6583 (53.0%)	1232/2665 (46.2%)	4722/9248 (51.1%)	< 0.01
**Infants Information**				
Gestational Age, Median (P25, P75)	30.0 (28.4,31.0)	29.7 (28.1,31.0)	29.9 (28.4,31.0)	< 0.01
< 26 weeks	221/6634 (3.3%)	121/2680 (4.5%)	342/9314 (3.7%)	< 0.01
26–27 weeks	848/6634 (12.8%)	412/2680 (15.4%)	1260/9314 (13.5%)	
28–29 weeks	2231/6634 (33.6%)	896/2680 (33.4%)	3127/9314 (33.6%)	
30–31 weeks	3334/6634 (50.3%)	1251/2680 (46.7%)	4585/9314 (49.2%)	
Birth Weight, Mean(SD)	1333.70 (323.21)	1294.63 (313.99)	1322.46 (321.06)	< 0.01
Male,N(%)	3832/6624 (57.9%)	1439/2679 (53.7%)	5271/9303 (56.7%)	< 0.01
Small for gestational Age,N(%)	546/6618 (8.3%)	165/2664 (6.2%)	711/9282 (7.7%)	< 0.01
Apgar score < 7 at 5 min,N(%)	480/6188 (7.8%)	178/2537 (7.0%)	658/8725 (7.5%)	0.23
Inborn,N(%)	4153/6634 (62.6%)	1779/2680 (66.4%)	5932/9314 (63.7%)	< 0.01

Data are presented in N (%), median (P25, P75) or mean (SD)

*ART* Assisted reproductive technology, *SD* Standard deviation

*P* value referring to the comparison between singleton and twin pregnancies

### Neonatal outcomes and NICU treatment

The NICU mortality rate was 14.1% for twins and 11.5% for singletons. Twins had higher overall incidences of composite outcomes (49.4% vs. 47.2%, *p* = 0.046) and short-term composite outcomes than singletons (74.4% vs. 68.3%, *p* < 0.01). Twins had significantly higher incidences of mortality (14.1% vs. 11.5%, *p* < 0.01), severe intraventricular haemorrhage (IVH) (8.9% vs. 7.4%, *p* = 0.02) and RDS (72.8% vs. 66.2%, *p* < 0.01) than singletons. However, there were no significant differences in NEC ≥ Stage II, BPD, severe neurological injury, cPVL, severe ROP, sepsis, or early death between these two groups (Table 2).

**Table 2:** Comparison of neonatal outcome in singleton and twin pregnancies

Outcome,N(%)	Singleton	Twin	Overall	*P* value
**N**	6634	2680	9314	
**Composite Outcome**	3129/6634 (47.2%)	1325/2680 (49.4%)	4454/9314 (47.8%)	0.046
Mortality	765/6634 (11.5%)	378/2680 (14.1%)	1143/9314 (12.3%)	< 0.01
NEC ≥ Stage II	354/6634 (5.3%)	133/2680 (5.0%)	487/9314 (5.2%)	0.46
BPD	2293/6612 (34.7%)	983/2673 (36.8%)	3276/9285 (35.3%)	0.057
Severe neurologic injury^a^	626/5599 (11.2%)	285/2245 (12.7%)	911/7844 (11.6%)	0.058
Severe IVH	412/5572 (7.4%)	198/2222 (8.9%)	610/7794 (7.8%)	0.02
cPVL	325/5836 (5.6%)	138/2348 (5.9%)	463/8184 (5.7%)	0.58
Severe ROP^b^	211/5138 (4.1%)	94/2038 (4.6%)	305/7176 (4.3%)	0.33
Sepsis	576/6337 (9.1%)	253/2542 (10.0%)	829/8879 (9.3%)	0.21
**Short-term Composite Outcome**	4529/6634 (68.3%)	1994/2680 (74.4%)	6523/9314 (70.0%)	< 0.01
Early Death	429/6612 (6.5%)	199/2673 (7.4%)	628/9285 (6.8%)	0.1
RDS	4355/6580 (66.2%)	1946/2672 (72.8%)	6301/9252 (68.1%)	< 0.01

Data are presented in N (%)

*NEC* Necrotizing enterocolitis, *BPD* Bronchopulmonary dysplasia, *IVH* Intraventricular hemorrhage, *PVL* Periventricular leucomalacia, *ROP* Retinopathy of prematurity, *RDS* Respiratory distress syndrome

*P* value referring to the comparison between singleton and twin pregnancies

^a^Incidence of IVH grade III and above or cPVL was calculated within infants who had neuroimaging results

^b^Incidence of ROP was calculated within infants who finished the ROP screening

Twins were more likely to have surfactant usage (singletons, 48.3%; twins, 55.5%) and be breastfed (singletons, 48.3%; twins, 55.5%) than singletons (*P* < 0.01). However, there were no differences in the length of NICU stay [median (IQR) for singletons, 45(33, 59); twins, 46(34, 60)], exclusive breastfeeding (singletons, 17.3%; twins, 17.2%) or the duration of invasive ventilation [median (IQR) for singletons, 4.5(2, 10); twins, 5(2, 11)] between the twin and singleton groups (Table 3).

**Table 3:** Comparison of neonatal intensive care unit (NICU) resource in singleton and twin pregnancies

NICU Resource	Singleton	Twin	Overall	*P* value
**Length of NICU stay,Median(P25,P75)**^a^	45 (33,59)	46 (34,60)	45 (33,59)	0.07
**Surfactant,N(%)**	3206/6634 (48.3%)	1487/2680 (55.5%)	4693/9314 (50.4%)	< 0.01
**breastfeeding,N(%)**	4441/6634 (66.9%)	1894/2680 (70.7%)	6335/9314 (68.0%)	< 0.01
**Exclusive breastfeeding,N(%)**	1146/6634 (17.3%)	461/2680 (17.2%)	1607/9314 (17.3%)	0.93
**Duration of invasive ventilation,Median(P25,P75)**^b^	4.5 (2,10)	5 (2,11)	5 (2,10)	0.1

Data are N (%) or median (P25,P75)

*P* value referring to the comparison between singleton and twin pregnancies

^a^Length of NICU stay was calculated within Infants who were survived to discharge

^b^Duration of invasive ventilation was calculated within infants with invasive ventilation

### Impact of twin births on study outcomes

After adjusting for potential confounders, overall, twins had higher odds of short-term composite outcomes (adjusted odds ratio [OR] 1.28, 95% confidence interval [CI] 1.09–1.50), RDS (adjusted OR 1.30, 95% CI 1.12–1.50), mortality (adjusted OR 1.28, 95% CI 1.10–1.49), and BPD (adjusted OR 1.10, 95% CI 1.01–1.21) than singletons. There were no significant differences between singletons and twins in the incidences of NEC ≥ Stage II, severe neurological injury, severe ROP, or early death (Table 4).

**Table 4:** Comparison of adjusted OR for neonatal outcome investigated, stratified by GA < 28 weeks and GA ≥ 28 weeks

**Outcome**	**Total**^c^	** < 28 wk**^c^	** ≥ 28wk**^c^
**Composite Outcome**	1.09 (1.00,1.20)	1.20 (0.89,1.61)	1.07 (0.97,1.18)
Mortality	1.28 (1.10,1.49)	1.40 (1.02,1.91)	1.24 (1.00,1.55)
NEC ≥ Stage II	0.82 (0.64,1.05)	0.65 (0.40,1.05)	0.87 (0.65,1.16)
BPD	1.10 (1.01,1.21)	1.13 (0.86,1.49)	1.10 (0.99,1.22)
Severe neurologic injury^a^	1.03 (0.87,1.22)	1.31 (0.95,1.81)	0.96 (0.79,1.18)
Severe IVH	1.06 (0.85,1.33)	1.36 (0.97,1.90)	0.97 (0.75,1.25)
cPVL	1.01 (0.78,1.31)	1.44 (0.88,2.36)	0.92 (0.67,1.26)
Severe ROP^b^	1.33 (0.99,1.80)	1.16 (0.68,2.00)	1.51 (0.93,2.45)
Sepsis	1.09 (0.91,1.30)	1.10 (0.74,1.63)	1.10 (0.91,1.33)
**Short-term Composite Outcome**	1.28 (1.09,1.50)	1.93 (1.21,3.08)	1.26 (1.07,1.48)
Early Death	1.13 (0.91,1.42)	1.41 (1.00,1.99)	0.98 (0.73,1.31)
RDS	1.30 (1.12,1.50)	1.59 (1.02,2.47)	1.29 (1.10,1.51)

*GA* Gestational age, *OR* Odds ratio, *NEC* Necrotizing enterocolitis, *BPD* Bronchopulmonary dysplasia, *IVH* Intraventricular hemorrhage, *PVL* Periventricular leucomalacia, *ROP* Retinopathy of prematurity, *RDS* Respiratory distress syndrome

^a^Incidence of IVH grade III and above or cPVL was calculated within Infants who had neuroimaging results

^b^Incidence of ROP was calculated within infants who finished the ROP screening

^c^Covariates were adjusted for gestational age, sex, SGA, birthplace at the infant level. Covariates were adjusted for maternal age, antenatal steroid usage, hypertension, diabetes, cesarean section, ART, primigravida status at the maternal level. To account for the level-3 NICU cluster effect, an exchangeable correlation matrix was used in the multiple GEE/ALR regressions

Analysis of the use of NICU resources showed that, after adjusting for potential confounders, twins had a higher incidence of surfactant usage (adjusted OR 1.22, 95% CI 1.05–1.41) and longer NICU stay (adjusted mean ratio 1.03, 95% CI 1.00–1.06) than singletons. However, there were no significant differences in breastfeeding, exclusive breastfeeding, or the duration of invasive ventilation between twins and singletons (Table 5).

**Table 5:** Comparison of adjusted OR for NICU resource investigated, stratified by GA < 28 weeks and GA ≥ 28 weeks

**Outcome**	**Total**^c^	** < 28 wk**^c^	** ≥ 28wk**^c^
	**OR**	**OR**	**OR**
**Surfactant**	1.22 (1.05,1.41)	1.32 (0.88,1.98)	1.20 (1.03,1.39)
**Breastfeeding**	1.05 (0.93,1.17)	0.80 (0.61,1.07)	1.12 (0.98,1.29)
**Exclusive breastfeeding**	0.97 (0.84,1.13)	0.90 (0.66,1.23)	1.00 (0.84,1.20)
**Outcome**	**Total**^c^	** < 28 wk**^c^	** ≥ 28wk**^c^
	**Mean Ratio**	**Mean Ratio**	**Mean Ratio**
**Length of NICU stay**^a^	1.03 (1.00,1.06)	1.00 (0.93,1.08)	1.03 (1.00,1.06)
**Duration of Invasive Ventilation**^b^	1.07 (0.95,1.20)	0.96 (0.81,1.15)	1.16 (1.02,1.33)

*OR* Odds ratio, *NICU* Neonatal intensive care unit

^a^Length of NICU stay was calculated within infants who were survived to discharge

^b^Duration of invasive ventilation was calculated within infants with invasive ventilation

^c^Covariates were adjusted for gestational age, sex, SGA, birthplace at the infant level. Covariates were adjusted for maternal age, antenatal steroid usage, hypertension, diabetes, cesarean section, ART, primigravida status at the maternal level. To account for the level-3 NICU cluster effect, an exchangeable correlation matrix was used in the multiple GEE/ALR regressionsssss

## Discussion

### Principal findings

This study was a national-level comprehensive assessment of care practices and health outcomes of 9314 VPIs admitted to 57 Chinese tertiary NICUs from January 2019 to December 2019. It served to fill a gap in our knowledge of Chinese neonatal perinatal characteristics and outcomes between twins and singletons. We found that twins had more adverse neonatal outcomes, including higher incidences of mortality, RDS, and surfactant usage, and a longer NICU stay than singletons.

### Maternal characteristics

Our findings were similar to those from other studies showing that twins received more antenatal steroids and were more likely to be delivered by caesarean section, be conceived by ART and be born at a tertiary perinatal centre [1, 5, 7–10]. Although our study found that twins conceived by ART are substantially higher than singletons (39.5% for twins, 7.3% for singletons), the mortality of twins was slightly higher than that of singletons. Helmerhorst et al. reported that singletons conceived by assisted reproduction have significantly worse perinatal outcomes than those not conceived by assisted reproduction, but this is not the case for twins [27]. An explanation could be the fact that twin pregnancy patients who underwent ART had more early obstetric visits and a higher socioeconomic class than those with spontaneous pregnancy [28]. In our study, we speculated that twin pregnancy patients who had undergone ART may have had more intensive pregnancy surveillance and more planned births in hospitals with NICUs compared with spontaneous twin pregnancy patients, thus decreasing the mortality rate of twins to a certain extent. The possibility of a higher caesarean section rate for twins is due to the higher rate of emergency caesarean section in very preterm twins, which was supported by previous studies [7]. Garg et al. also noted that twins were more likely to be delivered by emergency caesarean sections than singletons (twins, 29.3%; singletons, 16.9%) [7].

### Neonatal mortality

We found that twins had a higher rate of mortality than singletons, which was consistent with previously published reports [4, 5, 7–9] but contrary to the report written by Kalikkot Thekkeveedu R et al. [29]. In the current study, when infants were stratified into two groups, we also observed a higher perinatal mortality rate in the less than 28 weeks gestation group and the 28 weeks or more gestation group compared with that in singletons. Kalikkot Thekkeveedu R et al. published neonatal birth data of preterm and term singleton, twin, triplet and higher-order births from the United States during the years 2000, 2003, 2006, 2009, 2012, and 2016 and found that the adjusted odds for mortality were similar for twin births compared to singleton births. In this paper, the data included twins and singletons of all gestational ages, not only those less than 32 weeks gestation [29].

A previous study found that neonatal mortality was strongly correlated with gestational age, sex and a lack of prenatal steroid use, whereas ART helped to reduce the rate of mortality [7]. Other studies have shown that monochorionicity, growth discordance or intrauterine growth restriction and prematurity may be possible reasons for the higher mortality rate of twins [9]. Papiernik et al. found that same sex pairs had higher levels of mortality only below 28 weeks of gestation, whereas twins from same sex pairs with nondiscordant growth had outcomes similar to those of singletons [9]. However, data from 124 NICUs in the United States indicated that this difference between twins and singletons is driven primarily by the smaller twin’s weight. Although our study showed that very preterm twins were also less likely than singletons to be from pregnancies complicated by hypertension and SGA and more likely to have received prenatal corticosteroids, they still had higher rates of mortality. For these babies, we speculated that complications specific to twins, such as monochorionicity and growth discordance, may be responsible for the greater risk of adverse outcomes.

### Neonatal morbidities

Our results revealed that the risk of RDS was higher in twins than in singletons. Although twins had a higher exposure to antenatal steroids, twins received more surfactant than singletons. Similar observations have been made previously in large population-based studies from Canada, Australia, the USA and South Korea [7, 10, 30, 31]. A retrospective cohort study from Choi SJ et al. including 450 singletons and 117 twins reported that multiple courses of antenatal corticosteroids were associated with a significantly decreased risk of RDS in singleton pregnancies. However, the current standard dose or interval for antenatal corticosteroid administration in singletons did not reduce RDS in twins [31]. Therefore, we speculated that antenatal steroids might have less of an effect on twins than singletons.

Interestingly, there were no significant differences in the rate of BPD and length of NICU stay in the two groups, but after adjusting for multiple variables, twins had slightly higher rates of these two morbidity outcomes than singletons (adjusted OR 1.10 [1.01,1.21] for BPD, adjusted OR 1.03 [1.00,1.06] for length of NICU stay, respectively). In addition, we found that twins were more likely to have RDS, receive more surfactant, and have a higher rate of BPD. It was shown that the major factors of BPD are ventilator-induced barotrauma and volutrauma on the premature lung [16]. In our study, the higher rate of BPD was due to the longer duration of invasive ventilation at and after 28 weeks of GA in twins.

We found no significant difference in breastfeeding between the two groups in the multivariate model, although the rate of breastfeeding for twins was higher than that for singletons. This result is consistent with previous studies [32–34]. A national Spanish cohort study reported that multiple preterm infants were more likely to be fed by a combination of both breastfeeding and bottle-feeding than singletons (33% versus 26%) [32]. Antibiotic treatment at delivery, in vitro fertilization and prenatal steroids were associated with a decreased risk for shorter in-hospital breastfeeding durations [32]. In the present study, the rates of ART and prenatal steroid usage in twins were much higher than those in singletons, which may explain the slightly higher rate of breastfeeding in twins.

Available studies on outcomes of twins and singletons have reported conflicting results on the risk for severe IVH, NEC ≥ Stage II and severe ROP. Our data did not show any differences in these three morbidities between twins and singletons, which was supported by other large population and multicentre studies [7, 11]. However, some other studies showed inconsistent results [10, 35]. Friling and colleagues found that singletons with lower GA and BW had a significantly higher rate of advanced ROP (stages II-III) (30.2%) than twins (23.1%), not adjusting for possible risk factors associated with ROP, such as oxygen usage and ventilation [35]. Qiu et al. reported that twins had a lower incidence of ROP than singletons after adjusting for multiple variables for all infants with ROP, not only for those with severe ROP [10], which may be a possible explanation for the inconsistency in our study.

### Strengths and limitations

Our retrospective study included a large number of very preterm infants in China with a rigorous protocol for checking the completeness of recruitment. This study allows us to better ascertain the effect of plurality on outcomes, and we can counsel and prognosticate for our patients with our nationwide data.

Our study has several limitations. Our cohort was hospital-based, and it was from a select group of large tertiary NICUs with the highest level of neonatal care in China, which may not be representative of the general population. Pregnant women with foetal death in the delivery room and early pregnancy losses were not included in our study, so we may have underestimated the real mortality rate for each of these groups, which may have led to selection bias. Unfortunately, the database contains limited obstetric data (such as the reasons for delivery, the quality of prenatal care, illicit drug use, and other pregnancy complications of pregnant women), which may affect the neonatal outcomes of infants. However, we recognize this as a limitation of our work and chose information that was recorded completely and accurately for analysis. However, the mothers included in this study were predominantly from tertiary centres and may actually be at a higher risk than the more general population. Both zygosity and chorionicity have been suggested to affect the outcomes of twins. Monozygosity has been associated with higher rates of congenital anomalies, and chorionicity is related to the risk of growth discordance and growth restriction [9, 36, 37]; however, we did not examine these factors because we lack data regarding zygosity and chorionicity.

## Conclusion

Overall, our study suggests that very preterm twins are at an increased risk of mortality, RDS, and surfactant usage, and have a longer length of NICU stay compared with singletons. More attention should be given to early severe RDS treatment and respiratory management for twins, which may be helpful to decrease the mortality rate and hospital stay. Further studies adjusting for confounders such as chorionicity, dizygosity, cord anomalies and socioeconomic factors are needed to explore the long-term outcomes of twins.

## Data Availability

The original contributions presented in the study are included in the article, further inquiries can be directed to the corresponding authors.

## Abbreviations

ALR: Alternating logistic regression
AOR: Adjusted odds ratio
ART: Assisted reproductive technology
BPD: Bronchopulmonary dysplasia
CHNN: Chinese Neonatal Network
CI: Confidence interval
cPVL: Cystic periventricular leukomalacia
CXR: Chest X-ray
GA: Gestational age
GEE: Generalized estimating equation
IQRs: Interquartile ranges
IVH: Intraventricular hemorrhage
NEC: Necrotizing enterocolitis
NICUs: Neonatal intensive care units
RDS: Respiratory distress syndrome
ROP: Retinopathy of prematurity
SDs: Standard deviations
SGA: Small for gestational age
VPIs: Very preterm infants
